# Cellular nanomechanics derived from pattern-dependent focal adhesion and cytoskeleton to balance gene transfection of malignant osteosarcoma

**DOI:** 10.1186/s12951-022-01713-1

**Published:** 2022-11-24

**Authors:** Yongtao Wang, Nana Wang, Yingjun Yang, Yazhou Chen, Zhengguo Zhang

**Affiliations:** 1 Department of Urology, The First Affiliated Hospital of Zhengzhou University, Zhengzhou University, Zhengzhou, 450052 China; 2 School of Medicine, Shanghai University, Shanghai, 200444 China; 3 Department of Pediatrics, School of Medicine, Shanghai General Hospital, Shanghai Jiao Tong University, Shanghai, 200080 China; 4 Materials Institute of Atomic and Molecular Science, Shaanxi University of Science and Technology, Xi’an, 710021 China; 5 Medical 3D Printing Center, The First Affiliated Hospital of Zhengzhou University, Zhengzhou University, Zhengzhou, 450052 China; 6 Henan Institute of Advanced Technology, Zhengzhou University, Zhengzhou, 450003 China

**Keywords:** Malignant osteosarcoma, Cellular nanomechanics, Focal adhesion, Patterned cytoskeleton tension, Gene transfection

## Abstract

Gene transfection was supposed to be the most promising technology to overcome the vast majority of diseases and it has been popularly reported in clinical applications of gene therapy. In spite of the rapid development of novel transfection materials and methods, the influence of morphology-dependent nanomechanics of malignant osteosarcoma on gene transfection is still unsettled. In this study, cell spreading and adhesion area was adjusted by the prepared micropatterns to regulate focal adhesion (FA) formation and cytoskeletal organization in osteosarcoma cells. The micropattern-dependent FA and cytoskeleton could induce different cellular nanomechanics to affect cell functions. Our results indicated that transfection efficiency was improved with enlarging FA area and cell nanomechanics in micropatterned osteosarcoma. The difference of gene transfection in micropatterned cells was vigorously supported by cellular internalization capacity, Ki67 proliferation ability and YAP mechanotranduction through the regulation of focal adhesion and cytoskeletal mechanics. This study is an attempt to disclose the relationship of cell nanomechanics and gene transfection for efficient gene delivery and develop multifunctional nanomedicine biomaterials for accurate gene therapy in osteosarcoma cells.

**Supplementary Information:**

The online version contains supplementary material available at 10.1186/s12951-022-01713-1.

## Introduction

Gene transfection, which introduces exogenous genes into the targeted cells for gene therapy and cancer treatment, has attracted considerable attention due to the potential applications in gene knockout or overexpression, protein decoration, and cell “old-to-young” reprogramming [1–3]. It has also become the significantly advantageous approach to produce the tailor-made genomes and functional proteins [4]. In past almost 40 years, various transfection technologies have been well expanded to enhance transfection efficiency, mainly including viral-mediated and nonviral-mediated methods [5–9]. Normally, viral-mediated method is a powerful technique to operate durable transfection because of the straightforward combination between viruses and genomes [10]. However, viral vectors may cause the non-specific inflammatory reaction in vivo and even trigger tumor incidence [11, 12]. To circumnavigate these problems, nonviral-mediated methods including physical and chemical ones are able to overcome these problems and present some advantages, such as operational simplicity, size-free gene package, and relative biosafety [13, 14]. For example, physical methods of microinjection, electropotation and sonoporation have been proposed to achieve the targeting gene delivery for efficient transfection [15–18]. Whereas, these strategies need expensive and complex equipments, and also destroy the integrity of host cells, thus leading to low cell viability [19]. Chemical methods including cationic liposomes, cationic polymers and cationic compounds are widely used to evaluate cellular uptake and trafficking of exogenous nanoparticles based on the interaction of cell membrane and electric charges [20–24]. Nevertheless, chemical technologies usually cause low gene transfection efficiency and confine cell lines.

Besides efficient gene carrier materials and transfection techniques, cell nanomechanics is also considered as the important factor to allow exogenous genes to pass through cell membrane and be successfully expressed in cells [25, 26]. Cellular microenvironment can induce cellular nanomechanics and regulate transfection of exogenous genes, including substrate chemistry, matrix stiffness, viscosity and cell topography [27–30]. These biophysical cues will arrange focal adhesion formation and cytoskeletal structures to alter cellular nanomechanics [31]. Plenty of studies have been reported that cellular morphology mediates cytoskeletal nanomechanics and cell activity to affect cell behaviors, such as spreading and adhesion, division and differentiated fate, migration and endocytosis [32–36]. Although internalization of exogenous nanoparticles has been investigated on various micro/nano-patterned surfaces, the influence of micropattern-dependent nanomechanics of malignant tumors on gene transfection is not clear and uptake mechanism of exogenous genes is extremely desired to be explained.

Osteosarcoma is a highly malignant tumor originating from mesenchymal cells [37]. Cancerous mesenchymal cells in bone can rapidly expand to build tumor osteoid tissue and cause the death of human beings, especially children and adolescents [38, 39]. Until now, in addition to traditional treatments of surgery, chemotherapy and targeted drugs, gene-related therapy has been regarded as the most effective strategy to outstandingly cure malignant osteosarcoma in clinical applications [40, 41]. However, due to the existing fact in the difference of metabolism and metastasis in various microenvironments, the enhancement of transfection efficiency in malignant osteosarcoma still maintains a great challenge. In this work, the morphology of osteosarcoma was controlled by micropatterns and then transfected by lipid/plasmid lipoplexes to elucidate the influence of micropattern-dependent nanomechanics in tumor cells on gene transfection. This study will pave a bright way to understand the interaction between tumor nanomechanics and gene transfection.

## Results and discussion

### Characterization of mask and micropatterns

**Fig. 1:**
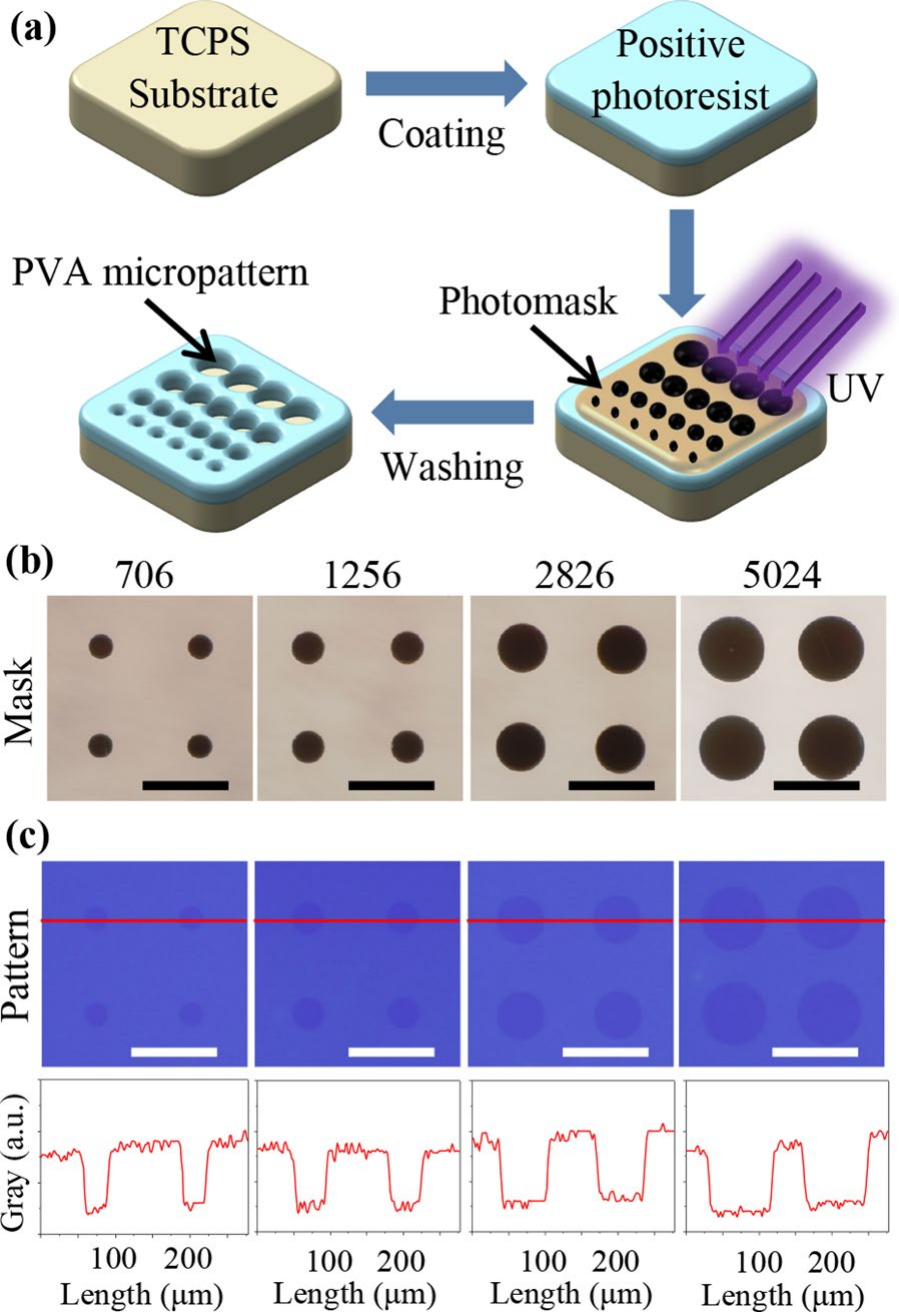
Preparation and characterization of designed mask and obtained micropatterns. **a** Schematic illustration of micropattern preparation through positive photoresist PVA on TCPS plates. **b** Representative images of designed mask with various microcircles. The diameters were designed as 30, 40, 60 and 80 μm and the corresponding spreading areas were 706, 1256, 2826 and 5024 μm^2^. Scale bar: 100 μm. **c** Representative images of prepared micropatterns captured by a fluorescence microscope for upper row. Scale bar: 100 μm. The bottom was the gray value of micropatterns calculated from the fluorescent images

The topological morphology of osteosarcoma was controlled by photolithography-induced micropatterns and then transfected by lipid/plasmid lipoplexes to elucidate the influence of micropattern-dependent nanomechanics on gene transfection in osteosarcoma cells. The photoreactive PVA (positive photoresist) is served as the protein-resistant biomolecules against cell adhesion [31]. The photoreactive PVA was homogenously overlaid to establish the nanoscale thin layer on TCPS and consequently micropatterned by the designed mask (Fig. 1a). The designed mask contained series of microcircles with different sizes (Fig. 1b). In specific, the diameters of the circles were defined to be 30, 40, 60 and 80 μm, and the corresponding spreading areas were calculated to be 706, 1256, 2826 and 5024 μm^2^. During the process of photolithography, short-wave UV light of 254 nm could pass through the transparent regions of the mask to crosslink the photoreactive PVA, yet not irradiate the dark regions of microcircles under the mask. The micropatterns were emerged by removing the uncrosslinked PVA of regions to manifest the exposed TCPS microcircles. The confirmation of micropatterns was observed by fluorescent microscope (Fig. 1c). The results demonstrated that the micropatterns were successfully transferred from the designed mask to the TCPS plates by photolithography. Furthermore, the images of micropatterns were analyzed by ImageJ software. There were different gray values in both patterned and non-patterned regions to distinguish the integrity of micropatterns. The gray values displayed the similar results in all patterned regions and indicated the uniformity of micropatterns. In addition, the diameters and spreading area were also measured based on the images of micropatterns and showed the same characters with designed ones (Additional file 1: Table S1). Therefore, the micropatterns could be controlled well through the synthesized PVA by photolithography.

### Cell micropatterning and FA formation

**Fig. 2:**
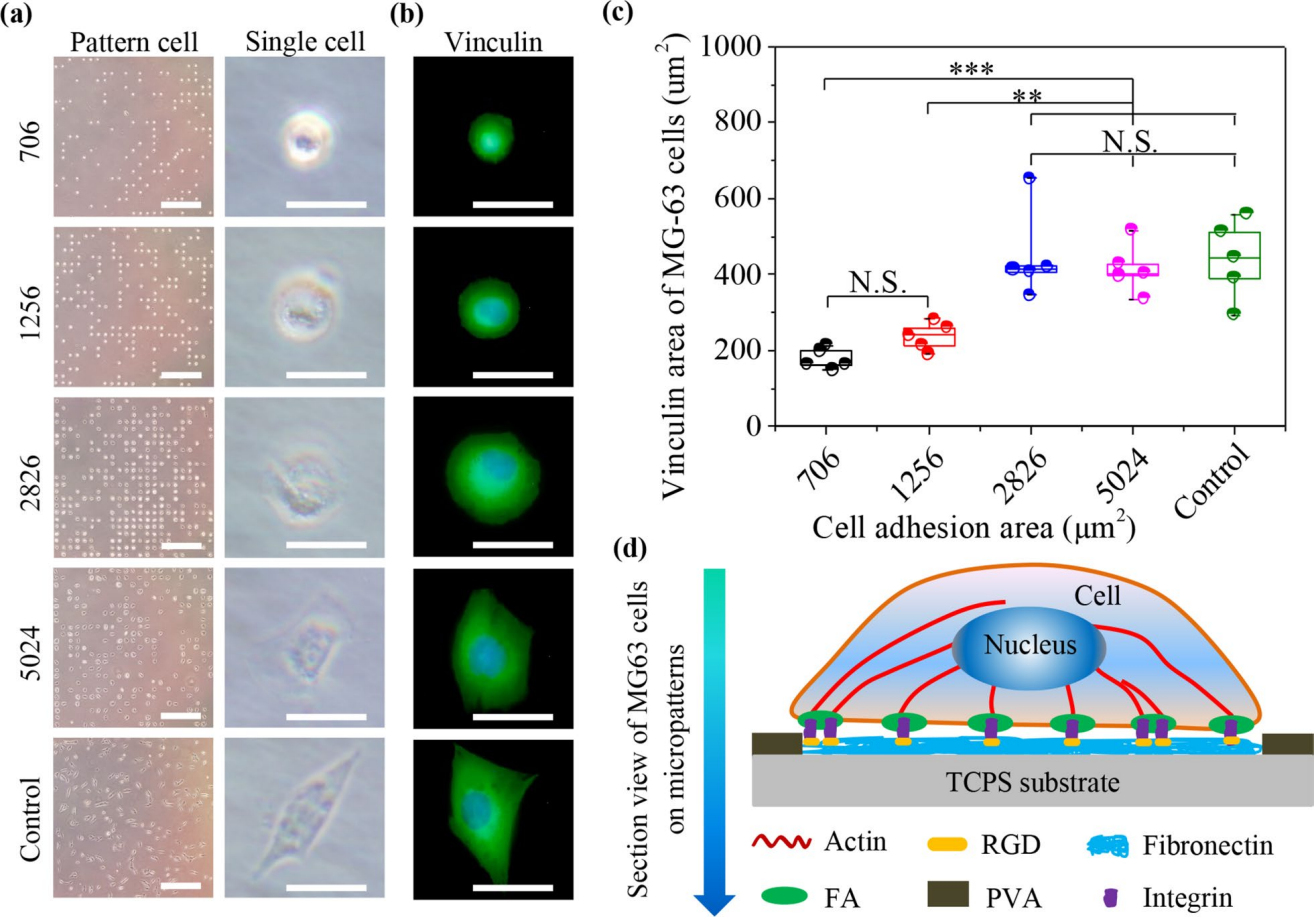
Cell micropatterning and formation of focal adhesion on micropatterned and non-patterned surfaces. **a** Representative microscopic images of MG63 cells cultured on four micropatterns and non-patterned surface. Scale bar: 1000 μm. The insets are the enlarged images to show the single cell morphology. Scale bar: 50 μm. **b** Representative immunofluorescent images of vinculin (green). The nucleus was stained blue with DAPI. Scale bar: 50 μm. **c** Vinculin area of MG63 cells calculated by ImageJ software. The data present the mean ± SD, n = 10, N.S. present no significance, **p < 0.01, ***p < 0.001. **d** Illustration to reveal focal adhesion serving as the signal bridge. Focal adhesion sensed microenvironmental stimuli from the micropatterns and transferred these biophysical signals into the cytoskeleton and even the nuclei to command cellular behaviors

MG63 cells were micropatterned on TCPS templates and the formation of focal adhesions was evaluated by vinculin staining. After MG63 cells were cultured on the micropatterned plates for 24 h, cell morphology was observed by an optical microscope (Fig. 2a). The cells were particularly attached onto the micropatterned areas, nevertheless, yet not to PVA-coated areas. The cell spreading area showed similar results with the designed area of micropatterns with 706, 1256 and 2826 μm^2^, while the cells could not spread their areas on the whole micropatterns with 5024 μm^2^ (Additional file 1: Table S2). The results indicated that cells could well accommodate to small micropatterns (706, 1256 and 2826 μm^2^), instead of large micropatterns (5024 μm^2^). It has been reported that oversize microcircles cannot well control cell morphology due to the limitation of inherent MG63 cell spreading area [31]. In addition, MG63 cells were also cultured on traditionally un-patterned TCPS surface as the control group. The cells of control group developed the spindle-shaped morphology on non-patterned surface.

Various cell morphologies can guide the formation and alignment of cellular attachment proteins [42]. Focal adhesion (FA) complexes that are tightly connected with cytoskeleton structures, act as the mechanosensor and transmitter during signal transduction [43]. FA kinase in FA sites is a fundamental ingredient of the signal delivery molecule within proximal cell membrane to administer extensive cell behaviors, including cell survival, spreading, division, differentiated fate, migration and endocytosis [44–46]. FA Kinase automatically maintains the dormant condition in mammalian cells, however, some biophysical cues from upstream microenvironment enables to recruit the activated kinase into FA complexes to adapt to external changes [47]. Vinculin, serving as a crucial component of FA complexes, can regulate cell adhesion functions [48]. Therefore, the formation of vinculin was investigated with immune-fluorescence staining in different micropatterned cells (Fig. 2b). The staining results presented that vinculin could be assembled in the whole cells, regardless of cell spreading area. Further, vinculin area of MG63 cells was calculated by the step-by-step approach (Fig. 2c). Vinculin area increased obviously with increasing cell spreading area from 706, 1256 and 2826 to 5024 μm^2^. Particularly, the cells with spreading area of 5024 μm^2^ formed twice vinculin area than that of 706 μm^2^. The vinculin area of non-patterned cells was calculated to be approximately 448 μm^2^, demonstrating the similar results with the 5024 μm^2^ cells. Some studies have reported that FA as the mechanotransductor will portray the signal bridge between integrin linkage and cytoskeleton structures [43]. The diverse signals derived from various FA structures have been confirmed and can affect cell spreading in malignant tumors [49]. The relationship was illustrated to reveal the interaction between FA and integrin, RGD or cytoskeleton (Fig. 2d). Focal adhesion sensed microenvironmental stimuli from the micropatterns and transferred these biophysical signals into the cytoskeleton and even the nuclei to command cellular behaviors, such as cellular internalization.

### FA-induced cytoskeleton to determine cellular nanomechanics in micropatterned cells

**Fig. 3:**
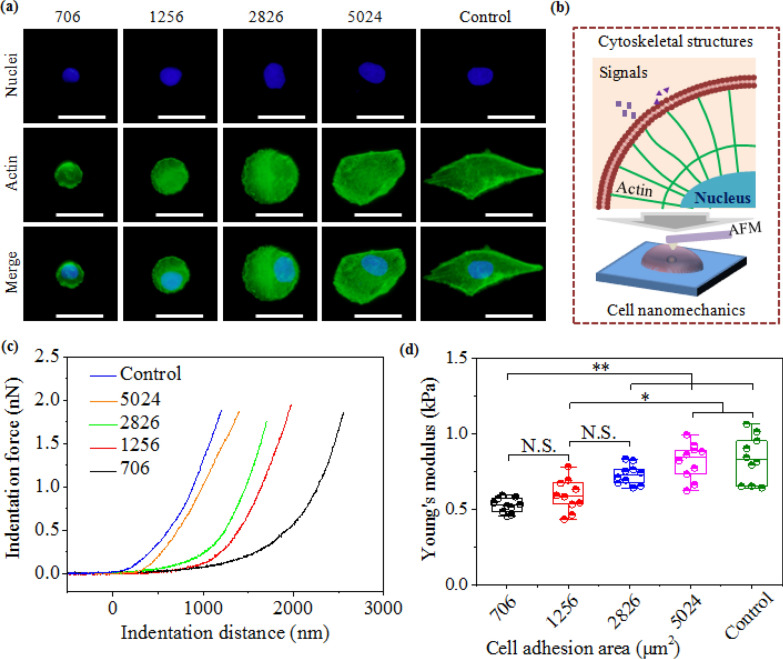
Cellular nanomechanics induced by cytoskeletal structures of micropatterned cells. **a** Fluorescent images of nucleus (blue) and actin bundles (green). Scale bar: 50 μm. **b** Illustration of cytoskeleton to affect cellular mechanics. Biophysical signals are induced into cells by mechanosensors on cell membrane to affect cytoskeletal assembly. The reorganization of actin bundles will regulate cell mechanics by the contractility. **c** Curves of indentation force and indentation distance measured by AFM. **d** Young’s modulus of micropatterned MG63 cells. The data present the mean ± SD, n = 10, N.S. present no significance, *p < 0.05, **p < 0.01

FA-induced cytoskeleton distribution and nanomechanics were analyzed by AFM nanoindentation. Microenvironmental cues are converted into intracellular signals by mechanosensing FA to induce the formation and alignment of cytoskeleton [50]. Actin is one of the basal components of cytoskeletal structures to support whole cell body [43]. Therefore, interconnected actin can produce intracellular nanomechanics due to convertible contractility to guide cell functions [51]. In this study, actin filaments were stained green in micropatterned cells (Fig. 3a). Cells assembled their actin filaments along the limited micropatterns in 706, 1256 and 2826 μm^2^ cells, while 5024 μm^2^ micropattern wasn’t filled with actin filaments. The cells on non-patterned surfaces formed the spindle-shaped network structures. Moreover, actin filaments developed fiber-like structures in 2826, 5024 μm^2^ cells and non-patterned cells, but disappeared in small cells (706 and 1256 μm^2^). As previously reported, stress fibers probably originate from radial fibers and transverse arcs by the interaction of cell-substrate interfaces. These emerging stress fibers may determine intercellular nanomechanics (Fig. 3b). To certify this hypothesis, an AFM nanoindentation method was applied to measure the indentation curves of force and distance to evaluate cellular nanomechanics (Fig. 3c). Further, cellular stiffness was analyzed by calculating the cellular stiffness of micropatterned and non-patterned cells (Fig. 3d). Young’s modulus presented gradually increasing tendency with enlarging cell spreading area, which was associated with the formation of stress fibers in well-spread cells. Therefore, the reorganization of cytoskeletal structures in micropatterned cells could affect cellular nanomechanics and implement the dynamic tension for internalization of exogenous genes, generating high gene transfection efficiency.

### Influence of micropattern-dependent nanomechanics on gene transfection of MG63 cells

**Fig. 4:**
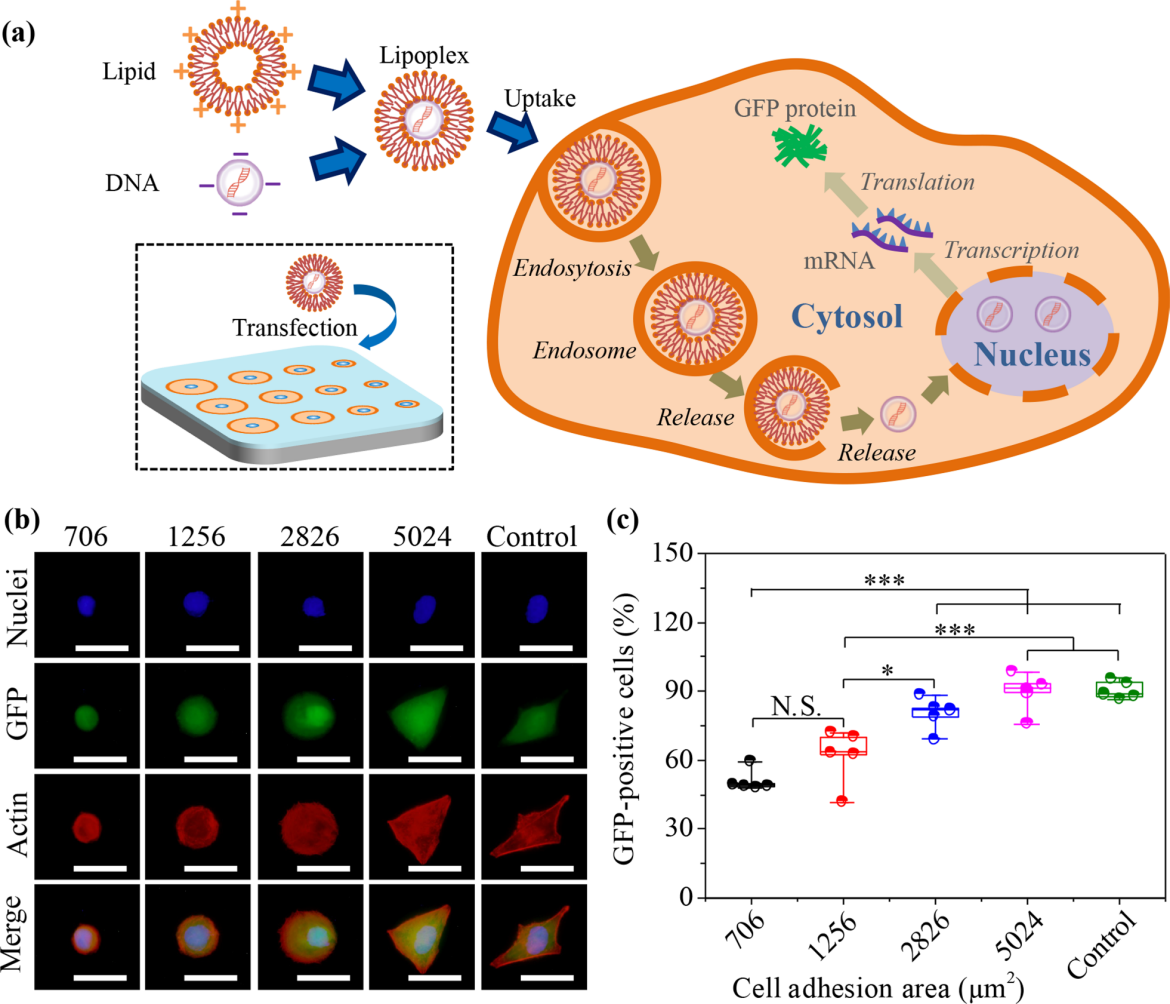
Influence of MG63 cytoskeleton on gene transfection in microcircles and non-patterned surface. **a** Illustration of gene transfection by incorporating plasmid with liposome into MG63 cells for intercellular delivery, nuclear transcription and GFP protein translation. **b** Representative fluorescence images of transfected MG63 cells expressing GFP proteins (green). Nucleus: blue; actin bundles: red. Scale bar: 50 μm. **c** Gene transfection efficiency by analyzing the amount of GFP-positive cells. The data present the mean ± SD, n = 5, N.S. present no significance, *p < 0.05, ***p < 0.001

Some stimuli, including biochemical stimuli (ECM, O_2_, growth factors and functional proteins) and biophysical stimuli (viscosity, stiffness, degradation and morphology) are important factors to govern gene transfection [52–54]. Therefore, the influence of micropattern-dependent nanomechanics of MG63 cells on gene transfection was disclosed on different microcircles. After gene transfection experiment was executed, the lipoplexes were internalized into cells by endocytosis and the plasmid would escape from the liposomes or lysosomes into nucleus for transcription and translation of GFP genes (Fig. 4a). The transfected MG63 cells expressing GFP proteins on the micropatterns were captured by a fluorescent microscope (Fig. 4b). Transfection efficiency was further calculated by analyzing the amount of GFP-positive cells in all checked cells (Fig. 4(c). Transfection results indicated that the percentage of GFP-positive cells was enhanced with enlarging MG63 cell adhesion area. The 706 μm^2^ cells showed the lowest transfection percentage (50.9%). Intriguingly, the 5024 μm^2^ cells and non-patterned cells showed similar transfection efficiency due to the consistency of cell adhesion area. The convergent results are confirmed to present the highest transfection efficiency in largest cell adhesion area of micropatterned hMSCs [55]. In addition, the micropatterns with larger size and aspect ratio enable to promote cell uptake capacity of gold nanoparticles [33]. Microscale pitted surfaces of 1–6 µm are also applied to disclose transfection mechanism of fibroblasts [56].

In addition of microscale patterns, nanoscale patterns and their composites are also prepared to regulate the effect of these aspects on gene transfection [57, 58]. Nanoscale grooves can induce different transfection efficiency of human lung fibroblasts by changing the width and height of nanogrooves [59]. A combination of micropillars (2 μm) and nanopillars (200 nm) is applied to adjust cellular topography, and this hierarchical structure will affect cellular internalization of dextran in hMSCs and COS7 cell lines [58]. Furthermore, biofunctional proteins are coated in the vertical arrangement of nanopillars to reveal their influences on transfection [29]. Exogenous genes are trapped into hollow nanotubes for efficient gene transfection. Therefore, these results may indicate that the alternation of geometric topography enables to determine gene transfection.

### Cytoskeletal nanomechanics to monitor cellular uptake capacity of FITC-SiO_2_ nanoparticles

**Fig. 5:**
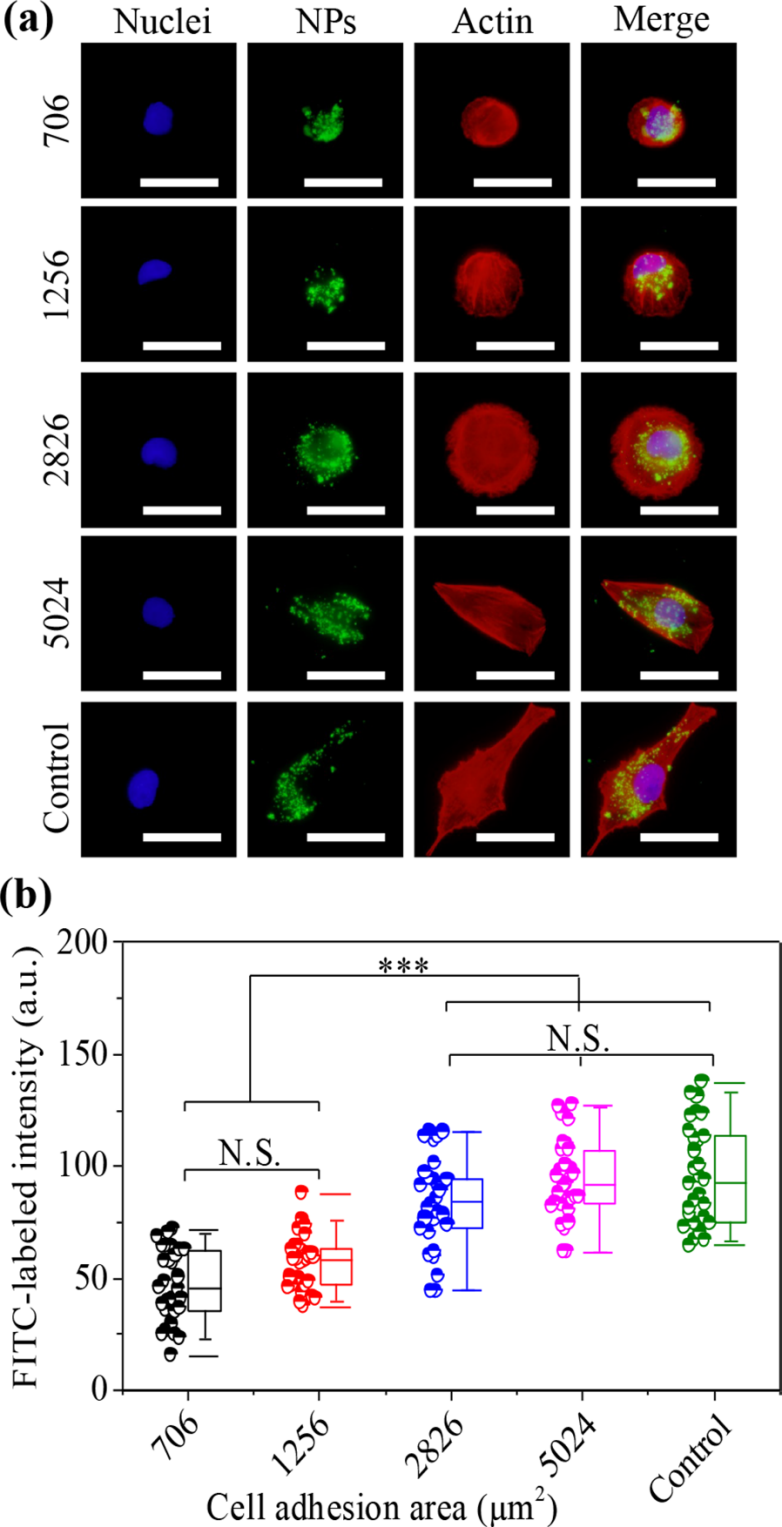
Regulation of cytoskeletal structures on cellular uptake capacity. **A** Fluorescent images of FITC-labeled microspheres (green). Nuclei: blue; actin: red. Scale bar: 50 μm. **B** Cellular uptake capacity by calculating the intensity of FITC-labeled NPs. The data present the mean ± SD, n = 10, N.S. present no significance, ***p < 0.001

As the first step of gene transfection, successful transmembrane delivery plays the decisive role in achieving highly efficient transfection [60]. Indeed, cell plasma membrane, as the natural semipermeable membrane can manipulate selective access to exogenous particles and deliver them into cells [61]. The internalization of nanoparticles (NPs) was explored by FITC-labeled NPs to declare cellular uptake capacity in micropatterned cells. The fluorescent images indicated that the amount of NPs increased with increasing cell adhesion area (Fig. 5a). Further, cellular uptake capacity was evaluated by the fluorescent intensity of FITC-labeled NPs in cells (Fig. 5b). The FITC-labeled intensity was improved with increasing cell adhesion area. The intensity of non-patterned cells showed similar results with 5024 μm^2^ cells. Cytoskeleton plays a decisive role in affecting cellular internalization capacity, which is associated with clathrin-mediated endocytosis [62]. As mentioned above, the reorganization of cytoskeleton in micropatterned cells could induce strong cell nanomechanics to stimulate high cell uptake capacity.

### Regulation of nuclear Ki67 activity and mechanotransduction by cytoskeletal nanomechanics

**Fig. 6:**
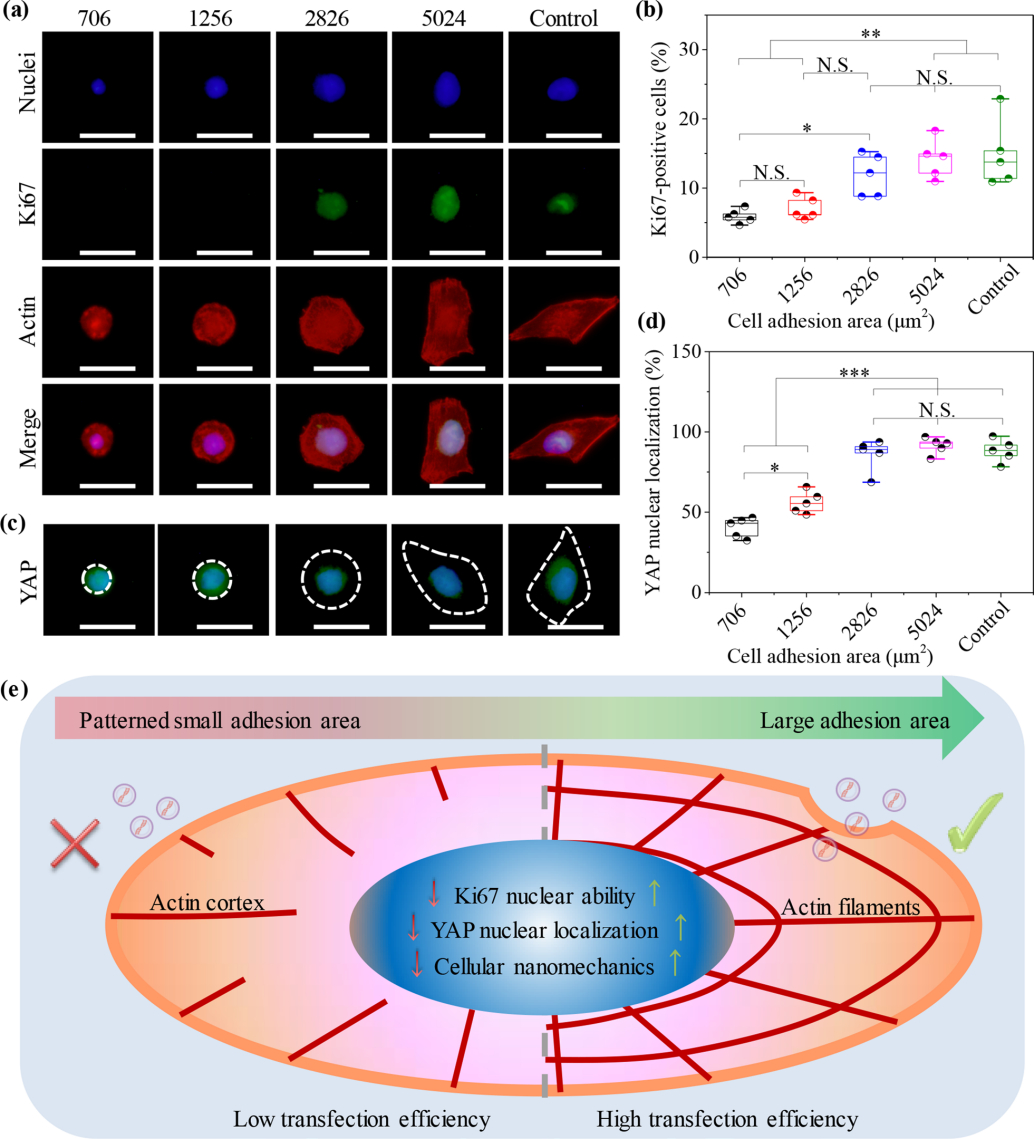
Influence of cytoskeletal structures on nuclear Ki67 activity and mechanotransduction. **A** Immunofluorescent images of Ki67 staining (green). Nuclei: blue; Actin: red. Scale bar: 50 μm. **B** Percentage of Ki67-positive cells in all checked cells. The data present the mean ± SD, n = 5, N.S. present no significance, *p < 0.05, **p < 0.01. **C** Immunofluorescent images of YAP staining (green). Nuclei: blue. White dotted circles present the outline of MG63 cells. Scale bar: 50 μm. **D** Percentage of YAP nuclear localization. The data present the mean ± SD, n = 5, N.S. present no significance, *p < 0.05, ***p < 0.001. **E** Illustration of micropattern-induced cytoskeleton nanomechanics to regulate gene transfection of MG63 cells

Nuclear Ki67 activity and mechanotransduction by cytoskeletal nanomechanics were regulated in micropatterned cells. Cytoskeleton-induced cell mechanics will affect cellular stemness and nuclear DNA synthesis on different substrates [32]. It has been reported that cell-morphology-dependent cytoskeleton can play a crucial aspect in regulating cellular behaviors, including cell adhesion, spreading, division, migration and uptake [30, 33]. Moreover, cell proliferation can enhance transfection of exogenous genes due to the disappearance of nuclear envelope during division process [63]. Exogenous genes are accessibly delivered into nuclei to participate in the procedures of the DNA synthesis, transcription and translation [64]. In this study, Ki67 staining was used to explore the influence of cytoskeletal nanomechanics on transfection efficiency. The staining results exhibited that Ki67 preferred to nuclear expression in larger cells, while nuclear Ki67 marker disappeared in 706 and 1256 μm^2^ cells (Fig. 6a). Furthermore, cell proliferation ability was analyzed by calculating the amount of Ki67-positive cells (Fig. 6b). The results disclosed that the percentage of Ki67-positive cells was enhanced with adjusting MG63 cell adhesion area from 706 to 5024 μm^2^. The cells on non-patterned surfaces presented the highest percentage of Ki67-positive cells because of the large adhesion area. The results disclosed that micropattern-induced cell mechanics could promote cellular proliferation ability on large micropatterns, which was beneficial for efficient gene transfection.

The reorganization of cytoskeleton is also converted to biochemical and bioelectrical signals in cytoplasm and subsequently transmitted into nuclei to affect cell functions [65]. Current studies have manifested that biomechanical signals regulate the highly positive expression of mechanics-related genes by altering cellular topography [31, 66]. Yes-associated protein (YAP) as the downstream of Hippo pathway plays the crucial role in controlling cell behaviors of tension-related genes [51]. Therefore, YAP and nuclei were co-stained to reveal the influence of cell adhesion area on cellular mechanotransduction (Fig. 6c). Because of the cytoskeletal difference induced by micropatterns, the expression of YAP gene showed different activity in micropatterned cells. Perceptibly, YAP was localized in nuclear regions when the cells had large adhesion areas, while YAP of small cells would disperse into cytoplasm. Further, the percentage of YAP nuclear localization was analyzed by calculating the amount of nuclear YAP-positive cells (Fig. 6d). YAP nuclear localization was enhanced with changing the adhesion area of MG63 cells, which showed the similar results with gene transfection. Therefore, cell morphology was controlled by micropatterns to investigate whether micropattern-controlled cell nanomechanics could affect gene transfection. Gene transfection showed a gradually rising tendency with enlarging cell adhesion area and nanomechanics on micropatterns. The transfection results were associated with the formation of focal adhesion, cellular uptake capacity, nuclear Ki67 activity and YAP mechanotransduction by the regulation of cytoskeleton-related nanomechanics (Fig. 6e).

## Conclusion

The topographical morphology of malignant MG63 cells was controlled by designed micropatterns through photolithography to induce different FAs and cytoskeletal structures. Cellular nanomechanics could be affected by the formation of micropattern-induced FAs and cytoskeleton structures. The micropatterned cells were transfected by lipid/plasmid lipoplexes to investigate their influence on gene transfection. Transfection results showed that the percentage of GFP-positive cells was notably enhanced with enlarging cell adhesion area, which could be explained by FA formation, cytoskeletal nanomechanics, high cell uptake capacity, cell division ability and YAP mechanotransduction. The results will make a deep understanding for the development of gene transfection in gene therapy and cancer treatment.

## Methods

### Mask design and micropattern preparation

The circle geometry was micropatterned onto the tissue culture polystyrene (TCPS) plates through photoreactive poly (vinyl alcohol) (PVA) by traditional photolithography method. The mask contained the dark circle micropatterns. The diameter of microcircles was designed to be 30, 40, 60 and 80 μm and the corresponding spreading areas were 706, 1256, 2826 and 5024 μm^2^, respectively. Photoreactive PVA (PhPVA) was synthesized by the Steglich reaction, as previously reported [33]. Then, PhPVA aqueous solution with a concentration of 0.3 mg mL^−1^ was dropped on TCPS plates to form a PhPVA thin layer in the dark. The mentioned-above mask was covered on the PhPVA-coated TCPS surfaces. After UV irradiation and washing in water bath, the micropatterns were successfully transferred from the pre-designed mask to the PhPVA-coated TCPS plates. The mask was observed by using an optical microscope. The images of micropatterned cells were taken by using a fluorescent microscope and further characterized to analyze the integrity of micropatterns by an ImageJ software.

### Cell culture

Human osteosarcoma cell line (MG63 cells) were purchased from Chinese Procell Lifer Science & Technology Co., Ltd. and subcultured in DMEM medium (Xi’an Mishu Biotechnology Co., Ltd., China) at 37 ℃ in a 5% CO_2_ incubator. The medium was supplied with 10% FBS (Sigma-Aldrich Co. LLC., USA) and 1% penicillin-streptomycin (Xi’an Mishu Biotechnology Co., Ltd.). Then, the MG63 cells were harvested from a 25 cm^2^ cell culture flask and diluted to form the cell suspension with 3 × 10^4^ cells/mL in cell density. Cell seeding experiment was carried out according to previous reports [30]. Before MG63 seeding on the micropatterns, the ethanol-sterilized TCPS plates were placed into the 6-well culture dishes and covered with a 1.5 × 1.5 cm^2^ PDMS frame with 0.1 mm thickness (Hangzhou Bald Advanced Materials Technology Co., Ltd., China) to avoid cell leaking in MG63 seeding. A 200 μL aliquot of cell suspension solution (6000 cells) was seeded into each PDMS frame. MG63 cells were cultured at 37 °C in a 5% CO_2_ incubator for 24 h to maintain the complete occupation of MG63 cells on micropatterns. An optical microscope was used to observe the morphology of MG63 cells on micropatterns. All experiments of cell culture were implemented in super-clean bench and the used tools were sterilized in an autoclave machine.

### Plasmid treatment

The plasmid (pAcGFP1-C1, Clontech Laboratories, Co. LLC., USA) expressed green fluorescent proteins (GFPs) in eukaryotic cells. The commercial plasmid was proliferated in Escherichia coli to obtain batch plasmid product, as previously reported [36]. Then, the purification of proliferated plasmid was executed by American Qiagen Plasmid Mini Kit based on the company's manners. The treated plasmid was analyzed by Nanodrop spectrophotometry to measure the concentration (Thermo Fisher Co. LLC., USA).

### Immunofluorescence staining of focal adhesion

After MG63 cells were incubated on micropatterned plates for 1 day, the samples were treated by 4% paraformaldehyde (Shanghai Aladdin Biochemical Technology Co., Ltd., China) and 1% Triton X-100 (Shanghai Aladdin Biochemical Technology Co., Ltd., China) for 10 min, handled with 0.02% Tween-20 for 30 min and blocked with 2% BSA (Sigma-Aldrich Co. LLC., USA) for 30 min. 1% Mouse anti-vinculin primary antibody (Merck KGaA, Germany) was used to incubate the micropatterned cells overnight. After that, the cells were stained with 1‰ Alexa Fluor 488-labeled anti-mouse secondary antibody for 1 h. Nucleus was stained with 1‰ DAPI in the dark for 10 min. The fluorescent images were observed by fluorescence microscope. Vinculin area of MG63 cells was calculated by using the ImageJ software to evaluate the formation of FA through a step-by-step method. Five independent experiments were applied to analyze the mean and SD.

### Cytoskeleton staining and stiffness measurement

After incubation for 1 day, the micropatterned MG63 cells were treated by 4% paraformaldehyde and 1% Triton X-100, blocked with 2% BSA. After 3 PBS washes, nucleus and actin bundles were co-stained with 1‰ DAPI and Alexa Fluor-488 phalloidin in the dark, respectively. The staining cells were observed by a fluorescence microscope. Furthermore, the living MG63 cells were measured by a silicon nitride cantilever fixed on an atomic force microscope (AFM) to obtain the curves of force and distance. Young’s modulus was calculated by the curves based on Hertz model method [31]. All cells were evaluated less than 2 h to keep cell living ability. Ten MG63 cells were applied to analyze the mean and SD.

### Transfection of MG63 cells

Transfection was executed by Lipofectamine 2000 (Lipo2000) (Invitrogen, USA). In specific, 1 μL Lipo2000 and 500 ng pAcGFP1-C1 were dissolved in 100 μL Opti-MEM (Life Technologies, USA), respectively. After 5 min incubation, the plasmid solution was added into Lipo2000 solution to prepare the cationic Lipo2000/plasmid lipoplexes. The cationic lipoplexes were further cultured for 30 min to maintain the stable structures. After MG63 cells were incubated on the micropatterns for 1 day, the DMEM was replaced by Opti-MEM for 2 h. Then, 200 μL cationic Lipo2000/plasmid lipoplexes was used to replace the Opti-MEM. The micropatterned cells were transfected in a 5% CO_2_ incubator for 6 h. Finally, Transfection solution was replaced by DMEM and the cells were further cultured for 18 h to express the GFP proteins. After gene transfection, the samples were treated by 4% paraformaldehyde. The fixed cells were handled with 1% Triton X-100 and blocked with 2% BSA. The nucleus and actin filaments of transfected cells were co-stained with 1‰ DAPI (Shandong Sparkjade Scientific Instruments Co., Ltd.) and Alexa Fluor-594 phalloidin (Invitrogen, USA), respectively. MG63 cells were observed by fluorescent microscope to determine whether they were transfected to GFP-positive cells. The fluorescent images were taken and further evaluated by the ImageJ to calculate gene transfer efficiency, as previously reported [36]. Five independent assays were used to analyze the mean and SD.

### Cell uptake of nanoparticles

Cell uptake capacity was analyzed by FITC-labeled SiO_2_ nanoparticles with a radius of 200 nm (Xi’an ruixi biological Technology Co., Ltd., China). First, Lipo2000 and nanoparticles solution were dissolved in Opti-MEM, respectively. After 5 min culture, the cationic Lipo2000/nanoparticle complexes were prepared by directly adding the nanopaticles into Lipo2000 solution and further incubated for 30 min. The uptake experiment was performed in the same process with gene transfection. After cellular uptake, the cells were covered with 0.4% trypan blue (Shanghai Aladdin Biochemical Technology Co., Ltd.) to quench the extracellular fluorescence of nanoparticles, and followed with PBS washing. Then, the MG63 cells were treated with paraformaldehyde and Triton X-100, blocked with BSA. After 3 PBS washes, nucleus of micropatterned cells and actin filaments were co-stained with DAPI and Alexa Fluor-594 phalloidin. Finally, the fluorescent images were recorded by a fluorescence microscope and analyzed by the ImageJ. The equation of *CFI* = *TFI-(A* × *AFI)* was used to calculate the correct fluorescence intensity (*CFI*) of micropatterned cells, *A* was cell area of interest, *TFI* and *AFI* were total fluorescent intensity of checked cells and average fluorescent intensity (background) of the micropatterned surfaces. *CFI* was defined as cell uptake capacity. 25 cells were measured to analyze the mean and SD.

### Immunofluorescence staining of Ki67 and YAP

Cell proliferation assay and nuclei mechanotransduction were evaluated by Ki67 staining and YAP staining. In brief, after the micropatterned MG63 cells were seeded and cultured for 1 day, the samples were rinsed with PBS and treated with 4% paraformaldehyde and Triton X-100. The cells were blocked with 2% BSA for 30 min. After 3 PBS washes, both of Ki67 and YAP staining were executed by the incubation with respective antibody, mouse anti-Ki67 primary antibody (Thermo Scientific CO. LLC., USA) for Ki67 staining and mouse anti-YAP primary antibody (Santa Cruz Biotechnology, USA) for YAP staining at 4℃ overnight. After 3 PBS washes, Alexa Fluor-488 anti-mouse antibody (1:1000 in PBS) was used as second antibody to stain the micropatterned cells. Additionally, nucleus and actin bundles were co-stained with DAPI and Alexa Fluor-594 phalloidin in the dark. The fluorescent images were captured by a fluorescence microscope. The percentages of divisive cells and YAP nuclei localization were analyzed by calculating the ratios of Ki67-positive and YAP-positive cells in all checked cells. Five independent assays were used to analyze the mean and SD.

### Statistical analysis

Statistical analysis was evaluated to calculate significant difference. The quantitative and fluorescent results were averaged to exhibit the mean ± SD. Significant difference was calculated by using one-way analysis of variance (ANOVA). The significant difference was considered when p < 0.05.

## Supplementary Information

**Additional file 1: Table S1.** Characters of prepared micropatterns. The data are calculated from 3 independent micropatterns. **Table S2.** Diameters and spreading areas of micropatterned and non-patterned MG63 cells. The data are calculated from five cells for each type micropatterns.

## Data Availability

The data that support the findings of this study are available from the corresponding authors upon reasonable request.
