# Supplementary material for: Cellular nanomechanics derived from pattern-dependent focal adhesion and cytoskeleton to balance gene transfection of malignant osteosarcoma
Source: J Nanobiotechnology. 2022 Nov 24;20:499. doi: 10.1186/s12951-022-01713-1 (PMC9694872; doi:10.1186/s12951-022-01713-1)
Supplement: Supplementary file 1 — Additional file 1: Table S1. Characters of prepared micropatterns. The data are calculated from 3 independent micropatterns. Table S2. Diameters and spreading areas of micropatterned and non-patterned MG63 cells. The data are calculated from five cells for each type micropatterns. [file 12951_2022_1713_MOESM1_ESM.doc]

Electronic Supplementary Material

| **Cellular nanomechanics derived from pattern-dependent focal adhesion and cytoskeleton to balance gene transfection of malignant osteosarcoma** |
| --- |
| Yongtao Wang^1,2†^ (**🖂**), Nana Wang^3†^, Yingjun Yang^4^, Yazhou Chen^5^ (**🖂**), Zhengguo Zhang^1^ (**🖂**)  *^1^ Department of Urology, The First Affiliated Hospital of Zhengzhou University, Zhengzhou University, Zhengzhou 450052, China*  *^2^ School of Medicine, Shanghai University, Shanghai 200444, China*  *^3^ Department of Pediatrics, Shanghai General Hospital, Shanghai Jiao Tong University, School of Medicine, Shanghai 200080, China*  *^4^ Materials Institute of Atomic and Molecular Science, Shaanxi University of Science and Technology, Xi’an 710021, China*  *^5^ Henan Institute of Advanced Technology, Zhengzhou University, Zhengzhou 450003; Medical 3D Printing center, The First Affiliated Hospital of Zhengzhou University, Zhengzhou University, Zhengzhou 450052, China*  *^†^ These authors contributed equally to this work.*  Supporting information to DOI 10.1007/s12274-****-****-* (automatically inserted by the publisher) |

|  |
| --- |
| Address correspondence to Yongtao Wang, yongtao_wang@shu.edu.cn; Yazhou Chen, yzchenbio@zzu.edu.cn; Zhengguo Zhang, zzg7181@126.com |

**Table S1.** Characters of prepared micropatterns. The data are calculated from 3 independent micropatterns.

| Designed area of micropatterns (μm^2^) | 706 | 1256 | 2826 | 5024 |
| --- | --- | --- | --- | --- |
| Designed diameter of microcircles (μm) | 30 | 40 | 60 | 80 |
| Measured diameter of microcircles (μm) | 29.8±1.1 | 41.7±0.7 | 60.9±2.4 | 80.3±1.9 |
| Designed area of microcircles (μm^2^) | 706 | 1256 | 2826 | 5024 |
| Measured area of microcircles (μm^2^) | 697.1±52.1 | 1365.9±46.2 | 2915.8±237.0 | 5062.1±244.7 |

**Table S2.** Diameters and spreading areas of micropatterned asnd non-patterned MG63 cells. The data are calculated from five cells for each type micropatterns.

| Designed area of micropatterns (μm^2^) | 706 | 1256 | 2826 | 5024 | Control |
| --- | --- | --- | --- | --- | --- |
| Measured diameter of MG63 cells (μm) | 29.4±1.6 | 38.9±0.7 | 59.2±0.8 | --- | --- |
| Measured spreading area of MG63 cells (μm^2^) | 682.1±73.7 | 1187.9±45.7 | 2756.3±76.6 | 3511.2±206.3 | 3603.1±269.5 |
